# Amino acid and bioactive signatures of yellowfin tuna loins: ocean-specific patterns across major fishing grounds

**DOI:** 10.3389/fnut.2026.1796270

**Published:** 2026-05-14

**Authors:** Mário A. G. Quaresma, Beatriz Vaz Rezende, João Silva-Almeida, Gonçalo Pereira, Helder Lúcio, Breixo Ventoso, Helena Gonçalves, Maria Leonor Nunes, Cristina Roseiro

**Affiliations:** 1 CIISA – Center for Interdisciplinary Research in Animal Health, Faculty of Veterinary Medicine, University of Lisbon, Lisbon, Portugal; 2 AL4AnimalS – Associate Laboratory for Animal and Veterinary Sciences, Lisbon, Portugal; 3 Universidad Católica San Antonio de Murcia, Murcia, Spain; 4 Frinsa Group, Ribeira, Coruña, Spain; 5 Technology and Innovation Unit, National Institute for Agricultural and Veterinary Research (INIAV, IP), Oeiras, Portugal; 6 CIIMAR/CIMAR-LA - Interdisciplinary Center of Marine and Environmental Research, University of Porto, Matosinhos, Portugal; 7 GeoBioTec – Geobiosciences, Geoengineering and Geobiotechnologies, NOVA School of Science and Technology, Campus de Caparica, Caparica, Portugal

**Keywords:** amino acid profile, anserine, carnosine, creatine, *Thunnus albacares*, total protein

## Abstract

**Background:**

Tunas are among the most valuable and consumed fish worldwide. In 2022, global tuna landings reached 8.3 million tonnes, with Yellowfin tuna (*Thunnus albacares*; YFT) representing 18.8% of the total, and ranking fifth among the most landed finfish. This study tested three null hypotheses: H01: Total protein content and the amino acid composition do not vary between geographic origin; H02: Bioactive compound profiles do not differ among regions; H03: There are no compositional differences that allow discrimination of tuna by oceanic origin.

**Methods:**

Sixty loins (20 per ocean) were analyzed for total protein content (Kjeldahl), AA profile (by fluorescence detection), and bioactive molecules (by HPLC-UV/Vis).

**Results:**

Protein content (25.3 g/100 g) and general AA composition confirmed H_01_, though 11 of 17 AAs differed (*p* < 0.05). Bioactive compounds were mostly similar (H_02_), with higher anserine and creatine in Indian Ocean fish. Essential AA indices remained high across samples.

**Conclusion:**

YFT loins showed consistent total protein content (25.3 g/100 g of edible portion), supporting a ‘high-protein’ nutritional claim, and confirming that protein content and overall amino acid (AA) profile do not differ significantly among specimens from different oceans (H01). Likewise, muscle bioactive compounds were generally comparable across origins (H02), indicating that AA signatures and bioactive compound levels cannot reliably trace geographic origin.

## 1 Introduction

Tunas represent one of the most commercially significant and extensively consumed fish groups across global markets. Their prominence in international fisheries is attributed to their high economic value, substantial catch volumes, and recognized nutritional benefits, making them a key foundation of the blue economy (1, 2).

In 2022, global landings of principal tuna species were estimated at approximately 8.3 million tonnes, according to the most recent FAO statistics (3). Skipjack tuna (*Katsuwonus pelamis*) dominated the 2022 global tuna catch, making up 36.9% of all tuna specimens and ranking as the third most harvested finfish species, while Yellowfin tuna (*Thunnus albacares*) was in second place accounting for 18.8% of all tuna catches and ranked fifth among the most caught finfish species. The market trade of the tuna species is divided into two broad categories consisting of fresh, canned, or frozen, largely based on loins which constitute up to 50% of the fish (4). The different tuna species are directed toward distinct markets. Bluefin tunas are primarily reserved for high-end sashimi and sushi markets, whereas Skipjack and Yellowfin tunas are largely utilized in the canning industry (5–7). Besides the species, fish size strongly influences its commercial value and its commercial use.

It is now well established that fish consumption has numerous benefits for human health and well-being (8–11). Different species of tuna are highly valued worldwide as food due to their nutritional value and sensory attributes. In addition, the diversity of technological processes that can be used in their processing, as well as their numerous culinary uses, make them a preferred choice in the seafood markets.

Tuna is the most consumed fish in the European Union (23.5 kg per capita; live weight equivalent) and the second most consumed in the United States (5.0 kg per capita) (12, 13).

Its delicate flavour and firm texture further enhance consumer acceptance (14–17). The chemical composition of tuna varies from species to species and within a single specimen is also influenced by the type of cut (loins, chunks, bellies, steaks, etc.) (14, 18). Tuna cuts differ in chemical composition, primarily due to variations in fat content, which also influence protein levels. Nevertheless, tuna loins are an excellent source of high-quality protein (21–25 g/100 g), providing all indispensable amino acids (19, 20). Moreover, they are more digestible than other animal proteins because of their lower connective tissue content (4). Apart from protein, loins are also a relevant source of long-chain n-3 polyunsaturated fatty acids, chiefly EPA (20:5n-3), DPA (22:5n-3) and DHA (22:6n-3) (15, 21), nutrients widely linked to cardiovascular and cognitive benefits (22).

*Thunnus albacares*, commonly known as Yellowfin tuna (YFT), is a highly migratory and widely distributed species in tropical and subtropical seas of the Pacific, Atlantic, and Indian Oceans, but generally absent from the Mediterranean Sea (23). It is worth noting that this species is currently subject to the most diversified commercial processing, including canned products, loins, steaks, sushi, and related products (14).

Environmental conditions and diet can influence the biochemical composition of fish muscle, even within a single species, as previously observed by our team in the Atlantic cod (*Gadus morhua*) (24). Although total protein content in tuna is generally conserved, the relative abundance of individual amino acids may reflect differences in metabolic rates, trophic interactions, and prey availability across different oceans. Similarly, bioactive compounds, such as peptides, antioxidants, and other functional molecules, can accumulate in variable amounts depending on diet and environmental stressors. These factors provide a mechanistic basis for expecting detectable differences in amino acid profiles and bioactive compounds among tuna from distinct geographic origins, which may also support partial discrimination by oceanic provenance.

Because protein level and amino acid profile support both nutritional quality and, potentially, geographic fingerprinting, they can be considered valuable metrics for quality evaluation and traceability purposes (24). Essential amino acids such as lysine, leucine and valine, for instance, support tissue growth, immune competence and metabolic regulation (25, 26).

Anserine, carnosine, and creatine are naturally occurring bioactive compounds widely distributed in the skeletal muscle of vertebrates and are therefore abundant in meat and fish products. Biochemically, carnosine is a dipeptide (β-alanyl-l-histidine), anserine is also a dipeptide methylated product of carnosine (β-alanyl-1-methyl-l-histidine), while creatine is a metabolite derived from amino acids (arginine, glycine and methionine). Several preclinical studies have shown that carnosine and anserine possess antioxidant, anti-inflammatory, and anti-aggregation properties, contributing to neuroprotective effects in animal models of Alzheimer’s disease, reviewed by Caruso et al. (27). On the other hand, creatine is known by its effective ergogenic aids for athletes, enhancing anaerobic energy capacity, reducing protein breakdown, and promoting increased muscle mass and physical performance. Recent studies suggest numerous potential therapeutic applications (28).

However, detailed comparative data on the amino acid and muscle bioactive profiles of YFT from distinct oceanic regions remains scarce Therefore, this study aimed to answer to three null hypotheses:

*H01*: Total protein content and the amino acid composition do not vary between geographic origin.

*H02*: Bioactive compound profiles do not differ among regions.

*H03*: There are no compositional differences that allow discrimination of tuna by oceanic origin.

## 2 Materials and methods

### 2.1 Sampling

Samples of 60 Yellowfin tuna (*Thunnus albacares*; YFT) loins, sourced from three major oceanic regions: Atlantic, Indian, and Pacific Oceans, with 20 samples collected from each (FAO fishing areas 34, 51, and 71, respectively). Data on specimens’ weights, catch season, and corresponding fishing areas are detailed in Table 1. Yellowfin tuna specimens were delivered frozen (temperatures below −20 °C) to Frinsa’s company frozen storage facilities (Frinsa, Coruña, Spain).

**Table 1:** Yellowfin tuna weight, fishing period and fishing areas according the Ocean of capture

**Ocean of Origin**	**Atlantic**	**Indian**	**Pacific**
Sample size (*n*)	20	20	20
Weight^1^	31.9 ± 5.1	51.6 ± 5.9	42.3 ± 12.6
Fishing periods	September–October	April–June	March–April
FAO fishing areas	34	51	71

^1^ It represents the whole yellowfin tuna weight, and it is expressed in kg and presented as mean ± standard deviation.

The 20 individuals from each oceanic region were randomly selected from the frozen stock, weighed, and thawed. The study focus, exclusively, on tuna loin. In anatomical terms, the loin corresponds to the epaxial muscle mass situated above the lateral line and extends along the dorsal region of *Thunnus albacares*, from the head to the caudal peduncle, after removal of the skin and bones. Herein, we used the cranial portion of the loin, collected from the dorsal area located between the head and the first dorsal fin (comprising exclusively white muscle tissue).

The samples were stored under vacuum and transported to the laboratory in a refrigerated environment. Each portion was then divided into three equal segments by cross-cutting along the craniocaudal axis and used for different analytical purposes. The anterior segments were used for determining protein content and amino acid composition. This portion was further sliced, finely chopped, and homogenized using a domestic food processor (Moulinex, France). The homogenate was kept refrigerated (below 5 °C) overnight and analysed on the following day.

### 2.2 Reagents

All general-purpose analytical-grade reagents, including hydrochloric acid, sodium acetate, sodium tetraborate, and 2-mercaptoethanol, were obtained from Merck Biosciences (Darmstadt, Germany). HPLC-grade ortho-phthalaldehyde, methanol, and tetrahydrofuran were sourced from Sigma Aldrich (St. Louis, United States), HPLC-grade acetonitrile and ammonium acetate were purchased from Merck (Darmstadt, Germany), and ultrapure water (Milli-Q) was also of HPLC-grade. Concentrated hydrochloric acid (37%) was supplied by Honeywell Research Chemicals (New Jersey, United States). Amino acid standards, comprising aspartic acid, asparagine, glutamic acid, glutamine, serine, histidine, glycine, threonine, arginine, alanine, tyrosine, valine, methionine, tryptophan, phenylalanine, isoleucine, leucine, and lysine, together with carnosine, anserine, and creatine standards, were all provided by Sigma Aldrich (St. Louis, United States).

### 2.3 Protein and amino acid analysis

The total protein content was assessed using the Kjeldahl method, following the guidelines established by the Association of Official Analytical Chemists (29). Each sample was analysed in triplicate, and for statistical purposes, the average of the two replicates showing the lowest coefficient of variation (always below 5%) was considered.

The amino acid (AA) profile was determined in duplicate using the method previously described by Aristoy and Toldrá (30), with slight adjustments as detailed by Quaresma et al. (31). Fluorescence detection was carried out at excitation and emission wavelengths of 338 nm and 425 nm, respectively. Identification of individual amino acids was achieved by matching retention times with those of known standards, and quantification was performed using an external standard calibration curve (peak area *vs*. concentration). The full amino acid composition is reported as milligrams per gram of edible tissue, while grouped amino acid values are expressed either as grams per 100 grams of protein and as milligrams per gram of edible portion.

### 2.4 Muscle bioactive compounds analysis

HPLC determination of carnosine, anserine and creatine were made according to Roseiro *et al.* (32). Approximately 8 g of each sample was homogenized with 15 mL of 0.01 N HCl (Polytron—PT3000, Kinematca Ag, Suisse) for 2 min and centrifuged at 10,000 rpm for 20 min at 4 °C. Supernatant was filtered through a glass fiber filter (Sartorius, Barcelona, Spain), and 250 μL of this solution was deproteinized by adding 750 μL of acetonitrile, standing at 4 °C for 20 min. The sample was then centrifuged at 10,000 rpm for 10 min at 4 °C and the supernatant filtered through Acrodisc 0.45 μm before HPLC analysis. The chromatographic separation was carried out in an Atlantis HILIC silica column (150 mm × 4.6, 3 μm) from Waters (Milford, MA) at room temperature. A linear gradient elution program was performed with a mixture of 0.65 mM ammonium acetate (pH 5.5) in water/acetonitrile (25:75) as solvent A and 4.55 mM ammonium acetate (pH 5.5) in water/acetonitrile (70:30) as solvent B. The gradient changed from 0 to 100% of solvent B in 13 min at a flow rate of 1.4 mL min^−1^. Separation was monitored using a photodiode array detector (PDA; Waters 996, Waters, Milford, MA) at 214 nm, and quantification was performed using the external standard method based on a calibration curve constructed from peak area versus analyte concentration.

### 2.5 Protein quality ratios and indices

The nutritional quality of YFT protein was evaluated using two key indices: the amino acid score (AAS) and the essential amino acid index (EAAI). The AAS was determined based on the World Health Organization’s (33) reference pattern for adults, which includes the following essential amino acid requirements in mg/g of protein: isoleucine (29), leucine (34), lysine (35), methionine plus cysteine (15), phenylalanine plus tyrosine (36), threonine (22), tryptophan (6), valine (37), and histidine (15).

The EAAI was calculated as the geometric mean of the ratios between the concentrations of essential amino acids in the tuna protein (His, Ile, Leu, Lys, Met+Cys, Thr, Trp, Val, and Phe + Tyr, expressed in g/100 g protein) and their corresponding values in the standard amino acid profile (*n* = 9). This index was expressed as a percentage. Both AAS and EAAI were computed accordingly the formulas proposed (38).


$$\mathrm{AAS}=\frac{\mathrm{mg}\;\text{of amino acid in}\;1\;g\;\text{of test protein}\;}{\mathrm{mg}\;\text{of amino acid in reference pattern}}\times 100$$

$$\text{EAAI}=100\times \sqrt[{n}]{\frac{\mathrm{Aa}1_{c}}{\mathrm{Aa}1_{\mathrm{rp}}}\times \frac{\mathrm{Aa}2_{c}}{\mathrm{Aa}2_{\mathrm{rp}}}*\ldots \times \frac{\mathrm{Aa}n_{c}}{\mathrm{Aa}n_{\mathrm{rp}.}}}$$

Where Aa refers to amino acids, the subscript “c” refers to the protein sample in analysis, “rp” to the reference pattern, and “n” to the number of essential amino acids.

### 2.6 Statistical analysis

In the results and discussion sections, the terms superiority and higher (expressed as a percentage) was defined as the ratio between the difference of the maximum and minimum values and the minimum value: (maximum−minimum)/minimum. All statistical analyses were conducted in two steps. The primary analytical procedure applied was the General Linear Model (Proc. GLM), which evaluated the effect of tuna origin (ocean) as a single factor. Canonical Discriminant Analysis (CDA) was also employed to explore potential group separations. Variable screening (significant effects in one-way ANOVA via Proc. GLM), interactive forward selection (Proc. STEPDISC), CDA, and cross-validation (Proc. DISCRIM) were all carried out in SAS 9.4 (SAS Institute Inc., Cary, NC, United States).

Statistical significance was determined at a threshold of *p* < 0.05. In cases where significant differences were identified, least square means were compared using Tukey’s test (*α* = 0.05) to control for multiple comparisons.

## 3 Results and discussion

### 3.1 Total protein and amino acid contents and amino acid profile

In this study, the total protein (TP) content of Yellowfin tuna loins ranged from 24.08 to 26.92 g per 100 g of edible portion (Table 2), slightly higher than the 23.0 g per 100 g reported for this species in the *Standard Tables of Food Composition in Japan* (39). Considering that the protein content in most fish species typically falls between 17 and 23 g per 100 g of edible tissue (40), these high TP levels support a “High Protein” claim, with protein providing at least 20% of the energy value (36).

**Table 2:** Total protein contents (expressed as g/100 g of edible portion), amino acid profile (expressed as mg/g edible portion) in Yellowfin tuna loin harvested in the Atlantic, Indian and Pacific Oceans

Amino acid	Ocean			Statistic	
	Atlantic	Indian	Pacific	SEM	*P*
Total protein	24.85	26.92	24.08	1.129	0.193
Indispensable amino acids					
Histidine	16.14^b^	26.28^a^	14.39^b^	2.479	0.002
Isoleucine	8.98^b^	6.99^b^	12.87^a^	0.946	<0.001
Leucine	13.62^a,b^	10.81^b^	14.51^a^	0.947	0.020
Lysine	22.32	18.99	25.01	2.336	0.198
Methionine	6.45	8.76	6.23	1.109	0.211
Phenylalanine	7.84^b^	7.18^b^	11.85^a^	0.891	<0.001
Threonine	15.23	16.43	13.21	2.241	0.592
Tryptophan	3.08	2.87	4.88	0.946	0.263
Valine	10.28	9.39	11.98	0.892	0.123
Conditionally indispensable amino acids					
Arginine	8.63^b^	13.63^a^	7.65^b^	1.213	0.002
Glycine	26.36^a,b^	30.72^a^	17.72^b^	2.968	0.010
Proline	5.48^c^	7.81^a^	6.76^b^	0.285	<0.001
Tyrosine	10.04^a^	4.37^b^	10.71^a^	1.638	0.015
Dispensable amino acids					
Alanine	4.20^a^	1.02^b^	3.16^a,b^	0.707	0.008
Aspartic acid	20.03	22.58	19.07	1.675	0.317
Glutamic acid	29.74^b^	39.14^a^	30.03^b^	2.619	0.020
Serine	21.01^a,b^	28.96^a^	15.28^b^	2.652	0.002

edible portion: fish muscle after removing bones, skin, fins;

Different superscripts in the same row are associated with significant differences (*P*<0.05); SEM – Standard error of the mean.

The high TP content observed in the loins of this species is also essential for its sensory characteristics, since, as a rule, species with low protein tend to lose more water during cooking, which impairs texture. The World Health Organization (WHO) recommends a daily dietary intake of protein of 830 mg protein/kg body weight for healthy adults; an additional 1, 9, and 31 g protein/day for pregnant women in the first, second, and third trimester, respectively; and 910 mg/kg body weight for children, in addition to specific recommendations for each of the indispensable amino acids. Based on this reference values and considering a 150 g serving for adults, the contribution of one serving of YFT to the recommended dietary intake (RDI) for a 65 kg adult would cover approximately 69% of that RDI.

The origin of the YFT had no significant effect on the TP content (*p* = 0.193) of fish caught of the Atlantic, Indian, and Pacific Oceans (Table 2).

The TP contents reported herein exceed those previously described for YFT (22.5–23.5 g/100 g) harvested in the Indian and Pacific Oceans (15, 37, 39, 41). In contrast, those specimens showed higher total lipid contents (1.93–2.46 g/100 g) than those previously analyzed in the specimens used in the present study (0.83–1.30 g/100 g), which have previously published (21). Given the well-established inverse relationship between intramuscular fat and protein concentration, these compositional differences likely contribute to the higher TP values observed in the present work.

Table 2 presents the concentrations of all amino acids. Among the indispensable amino acids (IAA), lysine, histidine, leucine, and threonine were found at the highest concentrations, whereas tryptophan showed the lowest concentration. These results are consistent with those of the *Standard Tables of Food Composition in Japan* (39). Ocean of origin significantly affected the histidine and leucine contents of YFT loins (*p* < 0.05), but had no effect on lysine or threonine contents (*p* > 0.05). YFT from the Indian Ocean exhibited higher histidine concentrations than those from the Atlantic and Pacific Oceans (72% higher), which did not differ significantly from each other (*p* > 0.05; mean = 15.3 mg/g edible portion). The highest and lowest leucine contents were observed in YFT harvested from the Pacific and Indian Oceans, respectively (14.5 vs. 10.8 mg/g edible portion), which differed significantly (*p* < 0.05). YFT from the Atlantic Ocean showed an intermediate leucine content (13.6 mg/g edible portion), which did not differ significantly from the other origins (p > 0.05).

Glutamic acid, which contributes to muscle flavour, was simultaneously the most abundant dispensable amino acid (DAA) and the most predominant of all AA in YFT loin. The YFT from the Indian Ocean exhibited a significantly higher glutamic acid content (31% higher) than those from the Atlantic and Pacific Oceans (*p* = 0.02).

Among the conditionally indispensable amino acids (CIAA), glycine was the most abundant.

The highest and lowest contents of glycine were observed among the YFT harvested in the Indian and Pacific Oceans, respectively, whereas the Atlantic YFT displayed a halfway value not differing significantly from the YFT harvested in the other oceans (*p* > 0.05). Our results do not support the hypothesis that higher glycine contents are associated with heavier YFT, even though heavier tuna species have been reported to contain higher collagen levels (42), which are correlated with glycine content.

The harvest origin significantly influenced (*p* < 0.05) the concentrations of 11 out of the 17 amino acids analysed, namely four IAAs (histidine, isoleucine, leucine, and phenylalanine), four CIAA (arginine, glycine, proline, and tyrosine) and three DAAs (alanine, glutamic acid, and serine). Pacific YFT were clearly higher in the levels of isoleucine (61.2%) and phenylalanine (57.8%) than Atlantic and Indian counterparts. It also showed higher leucine, tyrosine, and alanine compared to Indian YFT, but did not differ significantly from Atlantic samples. Conversely, the specimens from the Indian Ocean contained significantly greater amounts of histidine, arginine, proline, and glutamic acid when compared to samples from the other two regions. In addition, glycine and serine levels were higher in samples from the Indian Ocean than in those from the Pacific Ocean; however, their concentrations did not differ significantly from those in the Atlantic group (*p* > 0.05), which exhibited intermediate values for both amino acids.

The AA profile of YFT (Table 3) is mainly composed of IAA (103.96–114.93 mg/g of edible portion or 40.20–49.55 g/100 g of protein), followed by DAA (67.54–91.71 mg/g of edible portion or 26.95–34.01 g/100 g of protein), while CIAA accounted for the remaining content (42.83–56.54 mg/g of edible portion or 17.81–21.03 g/100 g of protein).

**Table 3:** Amino acids partial sums (expressed either as mg/g of edible portion and as g/100 g of protein) in Yellowfin tuna loin harvested in the Atlantic, Indian and Pacific Oceans

	Ocean			Statistic	
	Atlantic	Indian	Pacific	SEM	*P*
Partial sums (mg/g of edible portion)					
∑ TAA	229.46	255.96	225.31	10.43	0.088
∑ IAA	103.96	107.70	114.93	4.70	0.253
∑ CIAA	50.51^a,b^	56.54^a^	42.83^b^	3.55	0.029
∑ DAA	74.98^a,b^	91.71^a^	67.54^b^	5.42	0.008
Partial sums (g/100 g of protein)					
∑ TAA	92.96	95.25	94.31	1.730	0.647
∑ IAA	42.34^b^	40.20^b^	49.55^a^	1.749	0.001
∑ CIAA	20.71	21.03	17.81	1.220	0.131
∑ DAA	29.91^a,b^	34.01^a^	26.95^b^	1.346	0.002
Ratios					
TAA/TP	0.929	0.952	0.943	0.017	0.647
IAA/TAA	0.456^b^	0.423^b^	0.524^a^	0.016	<0.001
CIAA/TAA	0.225	0.220	0.188	0.012	0.071
DAA/TAA	0.322^a,b^	0.357^a^	0.287^b^	0.013	0.002

edible portion: fish muscle after removing bones, skin, fins.

Different superscripts in the same row are associated with significant differences (*P*<0.05); SEM – Standard error of the mean.

The evaluation of YFT loin shows that the origin had no effect on total IAA content (*p* = 0.253; averaging 108.86 mg/g of edible portion), but significantly influenced the total levels of CIAA and DAA (*p* < 0.05). Specifically, YFT from the Indian and Pacific Oceans exhibited the highest and lowest concentrations of both CIAA and DAA, whereas those from the Atlantic Ocean showed intermediate levels, which did not differ significantly from extreme values. The higher CIAA and DAA contents in Indian Ocean tuna relative to Pacific specimens were mainly driven by increased levels of arginine, glycine, proline, serine, and glutamic acid (*p* < 0.05).

However, significant differences were observed in IAA (*p* = 0.001) and DAA (*p* = 0.002). YFT from the Pacific Ocean exhibited the highest IAA content (49.55 g/100 g protein), whereas those from the Atlantic and Indian Oceans showed statistically similar values, averaging 41.27 g/100 g protein (*p* > 0.05). In contrast, the DAA fraction was highest in Indian Ocean YFT (34.01 g/100 g protein) compared to those from the Pacific Ocean (26.95 g/100 g protein), while the Atlantic samples presented intermediate values, not differing significantly from extreme values (*p* > 0.05).

Pacific YFT exhibited the highest ratio of indispensable amino acids to total amino acids (IAA/TAA), differing significantly (*p* < 0.05) from those from the Atlantic and Indian Oceans, which did not differ significantly from each other (*p* > 0.05). Conversely, Indian YFT showed a significantly higher ratio of dispensable amino acids to total amino acids (DAA/TAA) compared to those from the Pacific Ocean (0.357 vs. 0.287; *p* < 0.05), while the Atlantic counterparts showed intermediate values, showing no significant difference from either of the other two origins (*p* > 0.05). Additionally, the ratio of conditionally indispensable amino acids to total amino acids (CIAA/TAA) did not differ significantly among origins (*p* = 0.071).

**Figure 1:**
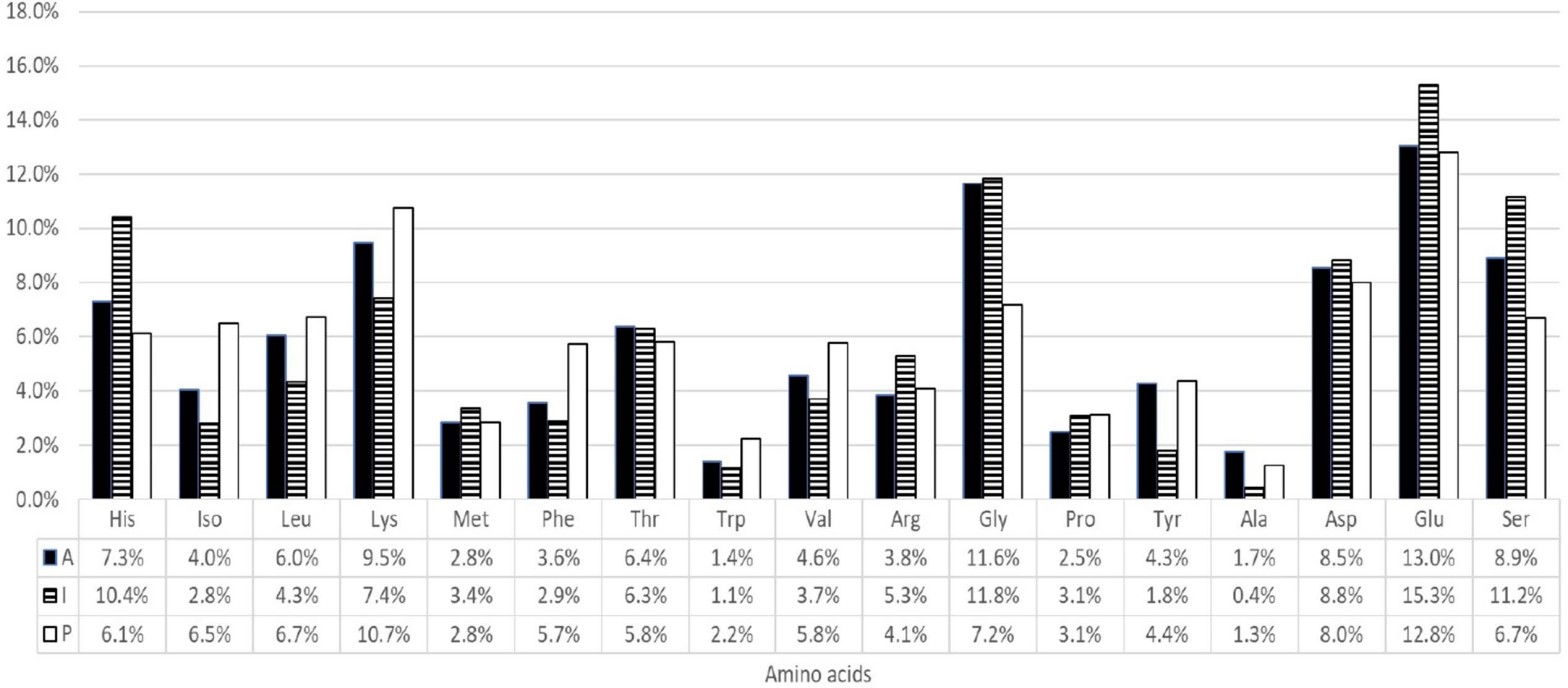
Amino acid profile (expressed as % of total amino acid content) in Yellowfin tuna loins harvested in the Atlantic (A), Indian (I), and Pacific (P) Oceans.

Figure 1 illustrates the amino acid profile (expressed as a percentage of total amino acid content) of YFT harvested in the three Oceans. The glutamic acid, a DAA, was the predominant component across all oceanic origins, representing between 12.8 and 15.3% of TAA. Glycine, a CIAA, ranks second, contributing with 7.2 to 11.8% of TAA, followed closely by lysine, an IAA, which accounts for 7.4 to 10.7% of the total. Altogether, the three most abundant amino acids comprised approximately 30.7 to 34.6% of the total amino acid content in YFT loin. Notable regional differences were observed: Indian Ocean samples exhibited the highest levels of glycine (15.3%) and histidine (10.4%), while Pacific samples had the highest lysine (10.7%) and leucine (6.7%) contents. Atlantic samples showed intermediate values for most amino acids. These findings highlight region-dependent variations in the amino acid composition of YFT loins.

At the other end of the spectrum, alanine and tryptophan were the least abundant, representing only 0.4 to 1.7% and 1.1 to 2.2% of the TAA, respectively.

The amino acid composition of fish muscle proteins is generally considered relatively conserved because it reflects the structural requirements of muscle proteins. Previous studies have reported that the overall amino acid pattern of fish muscle remains remarkably constant between populations within a single species and even across species and, with only minor quantitative variations (43, 44).

Nevertheless, the amino acid composition of fish muscle is dynamic and influenced by a range of biological and environmental factors, including water temperature, salinity, prey availability, and trophic interactions, all of which can vary across capture regions (35, 45, 46). The YFT is an opportunistic predator that feeds on a diverse array of prey species. Variations in diet composition can drive metabolic shifts, while ocean currents with differing temperatures may further impact metabolic processes, particularly those related to thermoregulation.

In tuna muscles, amino acids serve as structural components of three main protein classes: (1) myofibrillar proteins (structural and contractile proteins within muscle fibres); (2) sarcoplasmic proteins (water-soluble proteins, mainly enzymes and myoglobin) involved in metabolic processes; (3) stromal proteins – structural, water-insoluble proteins in the muscle’s connective tissue, predominantly composed of collagen, elastin, and minor proteins (47–49).

Myofibrillar and stromal proteins are the prime structural components of the loin muscle, associated with muscle fibres and connective tissue, respectively. Despite YFT epipelagic condition, typically inhabiting the upper 200 m of the water column, it often undertakes deep dives exceeding 500 m. This behaviour demands a range of physiological and structural adaptations to cope with elevated hydrostatic pressures and colder temperatures, as previously observed in *Thunnus obesus* (50). The frequency of such deep-diving events may, in turn, affect muscle composition by shifting the relative abundance of myofibrillar and stromal proteins. On the other hand, both myofibrillar and stromal proteins are encoded by the nuclear genome, regional differences in amino acid composition of tuna loins may also reflect underlying genetic divergence, expressed through variations in the amino acid sequences of these structural proteins. This interpretation is consistent with previous findings (51), who reported significant genetic differentiation between Atlantic and Indo-Pacific populations (51). Similarly, genetic divergences where observed between northern and southern equatorial YFT populations in the eastern Pacific (52), and three genetically distinct groups were also identified in the Indian Ocean (53).

In contrast, sarcoplasmic proteins are encoded by both the nuclear and mitochondrial genomes. Their contribution to regional differences in YFT loin amino acid profiles may be attributed to: (1) differences in mitochondria densities; (2) epigenetic regulation of transcriptional activity; (3) variations in metabolic pathways and associated enzyme expression; or (4) broader genetic background differences.

### 3.2 Amino acid score (AAS) and essential amino acid index (EAAI)

Protein quality is determined by how well it meets age-specific needs for essential amino acids and nitrogen to support growth, maintenance, and physiological functions. It depends on the protein content, the composition of essential amino acids, and their bioavailability (54). Herein we used two amino acid–based protein quality indices: (1) amino acid score (AAS) and (2) essential amino acid index (EAAI). Since both quantify how closely the protein’s amino acid profile matches human requirements, and provide complementary information.

Table 4 shows the amino acid scores (AAS) and the essential amino acid index (EAAI), to support the comparative nutritional assessment of YFT samples collected from different oceanic regions. The harvesting origin significantly influenced (*p* < 0.05) the AAS values of most essential amino acids, with the exceptions of methionine, threonine, and tryptophan, which showed average values of 129.9, 246.9, and 250.0, respectively. YFT harvested in the Indian Ocean displayed notably higher AAS for histidine (*p* = 0.003) compared to those from the Atlantic and Pacific Oceans, which did not differ statistically from each other.

**Table 4:** Amino acid Scores (AAS) and the Essential amino acid index (EAAI) in Yellowfin tuna loin harvested in the Atlantic, Indian and Pacific Oceans

AAS	Ocean			Statistic	
	Atlantic	Indian	Pacific	SEM	*P*
Histidine	435.4^b^	652.4^a^	373.0^b^	57.54	0.003
Isoleucine	127.8^b^	89.0^b^	208.4^a^	22.23	0.001
Leucine	96.7^a,b^	70.4^b^	108.6^a^	8.529	0.008
Lysine	197.6^a,b^	157.8^b^	225.3^a^	16.09	0.016
Methionine	122.6	144.7	122.4	19.97	0.666
Phenylalanine	195.3^a,b^	117.5^b^	253.1^a^	18.00	<0.001
Threonine	249.1	257.6	234.1	31.19	0.865
Tryptophan	221.0	179.7	349.2	63.47	0.153
Valine	109.8^a,b^	90.94^b^	141.0^a^	13.39	0.035
EAAI	152.9^a,b^	135.7^b^	167.4^a^	6.970	0.009

Different superscripts in the same row are associated with significant differences (*P*<0.05); SEM – Standard error of the mean.

The highest AAS values for leucine, lysine, phenylalanine, and valine were observed in specimens from the Pacific Ocean. Whereas, the lowest values for these amino acids were found in specimens from the Indian Ocean.

While the Atlantic Ocean counterparts had intermediate AAS for these amino acids, with no significant difference from the extremes. Furthermore, specimens from the Pacific Ocean exhibited significantly higher AAS for isoleucine than those from both the Atlantic and Indian Oceans (208.4 versus 108.4; *p* = 0.001). According to the World Health Organization reference pattern, all essential amino acid requirements were met or exceeded by YFT from the Pacific Ocean, and no limiting amino acid was detected (33). Conversely, Atlantic YFT exhibited a single limiting amino acid, leucine, which provides 96.7% of the daily recommended intake for adults. Whereas, the Indian specimens had the least favourable results, showing three limiting amino acids: valine (90.9%), isoleucine (89.0%), and leucine (70.4%). These data further substantiate the concept that geographic origin may affect the nutritional value of fish muscle, due to different contents in IAAs. Such result was previously observed in the Atlantic cod (*Gadus morhua*), between specimens harvested in the Exclusive Economic zones (EEZ) of Norway and Iceland (24).

The YFT origin influenced significantly the EAAI (*p* = 0.009). The specimens harvested in the Pacific and Indian Oceans presented the highest and lowest EAAI, differing significantly between each other (167.4 versus 135.7; *p* < 0.05), while the Atlantic Ocean YFT displayed a halfway value, not differing significantly from extreme values (*p* > 0.05). Despite the influence of origin on the EAAI, all three sources exhibited values above 100%, thereby qualifying as matching quality, the highest classification achievable for EAAI.

The results obtained from the EAAI are in line with those of AAS. The highest EAAI value, observed in the Pacific YFT was associated with the absence of limiting EAAs, while the lowest EAAI value, detected in the Indian YFT, was associated with three limiting EAAs. Whereas, the Atlantic YFT presented a midway value of EAAI and just one limiting EAA.

However, it is important to highlight that observed differences between oceans in the AAS or in the EAAI reflect compositional variations rather than substantial differences in protein nutritional value, since these indexes due not account differences in digestibility between AAs.

### 3.3 Muscle bioactive compounds

In addition to its amino acid profile, the YFT loin contained three physiologically relevant bioactive compounds, namely anserine, carnosine, and creatine (Table 5). These were quantified to substantiate its nutritional value and to explore their utility as discriminatory biomarkers. Biochemically, carnosine (β-alanyl-L-histidine) and anserine (β-alanyl-Nπ-methyl-L-histidine) are dipeptides, whereas creatine (aminoiminomethyl)-N-methylglycine is a metabolite derived from amino acids. For simplicity, all three are hereafter referred to as bioactive molecules. Carnosine and anserine play crucial roles in protecting cellular structures and maintaining fish muscle quality through their dual function as metal chelators and radical scavengers. By preventing oxidative deterioration, they help preserve cellular integrity as well as the sensory and nutritional properties of edible tissues (55, 56). Whereas, creatine is crucial for energy metabolism in the brain and skeletal muscle (27).

**Table 5:** muscle bioactive compounds in in Yellowfin tuna loin harvested in the Atlantic, Indian and Pacific Oceans (expressed as mg/100 g of edible portion)

	Ocean			Statistic	
	Atlantic	Indian	Pacific	SEM	*P*
Anserine	307.3^b^	367.6^a^	342.9^a,b^	13.91	0.012
Carnosine	25.62	27.08	27.91	2.056	0.730
Creatine	485.3^b^	569.4^a^	571.3^a^	19.39	0.003

edible portion: fish muscle after removing bones, skin, fins.

Different superscripts in the same row are associated with significant differences (*P*<0.05); SEM – Standard error of the mean.

The geographic origin of the YFT significantly influenced the concentrations of anserine and creatine (*p* < 0.05). Specimens caught in the Indian and Atlantic Oceans displayed the highest and lowest levels of anserine (367.6 and 307.3 mg/100 g of edible portion, respectively), differing significantly from each other but not significantly from those in the Pacific Ocean (342.9 mg/100 g). Regarding creatine, the highest concentrations were found in tuna from Indian and Pacific Oceans, averaging 570.4 mg/100 g of edible portion. These values were statistically similar (*p* > 0.05), but significantly exceeded those recorded in Atlantic Ocean specimens, which had a mean creatine content of 485.3 mg/100 g (*p* < 0.05). No significant differences were observed on carnosine levels among the different regions (*p* = 0.730), averaging 26.87 mg/100 g. In mammals and birds, the three bioactive molecules are found in higher concentrations in white (fast-twitch, type II) muscle fibres than in red (slow-twitch, type I) muscle fibres (32, 34, 57–60). The muscle fibres in YFT loin are classified as white muscle cells (61), sharing similar metabolic and biochemistry features with muscle fibres from mammals and birds. Within the same species, white muscle fibres in fish contain higher levels of anserine, carnosine, and creatine compared to those of corresponding red muscle (62).

### 3.4 Discriminatory ability of tuna amino acid profile and bioactive compounds

Given their high commercial value and global trade, tuna require robust traceability methods to ensure consumer protection and the sustainability of stocks (63). To assess the ability of amino acid profiles and muscle bioactive compounds to discriminate Yellowfin tuna loins by harvest region, a canonical discriminant analysis (CDA) was conducted. CDA revealed two significant functions (depicted in Table 6), with Root 1 explaining 59.41% of the variance and Root 2 the remaining 40.59% (*p* < 0.0001). The corresponding eigenvalues were 1.0131 (Root 1) and 0.6922 (Root 2), with canonical correlations of 0.7009 and 0.6396, respectively. Proline, isoleucine, and arginine contributed positively to Root 1, whereas phenylalanine showed a negative loading. Root 2 was mainly defined by the positive loadings of proline, creatine, and phenylalanine. Creatine was the only muscle bioactive compound with significant discriminant power, contributing to both Roots.

**Table 6:** Results of the canonical discriminant analysis: loadings of correlation matrix between predictor variables (standardized canonical coefficients) and discriminant functions (Roots 1 and 2), and some statistics for each function.

Variables	Root 1	Root 2
Proline	0.610081	0.666838
Isoleucine	0.627212	0.387879
Arginine	0.624916	0.031386
Creatine	0.181978	0.639098
Phenylalanine	-0.498207	0.481387
Statistics		
Canonical R	0.7009411	0.639569
Eigenvalue	1.0131	0.6922
Cumulative proportion	0.5941	1.000
Probability	<0.0001	<0.0001

**Figure 2:**
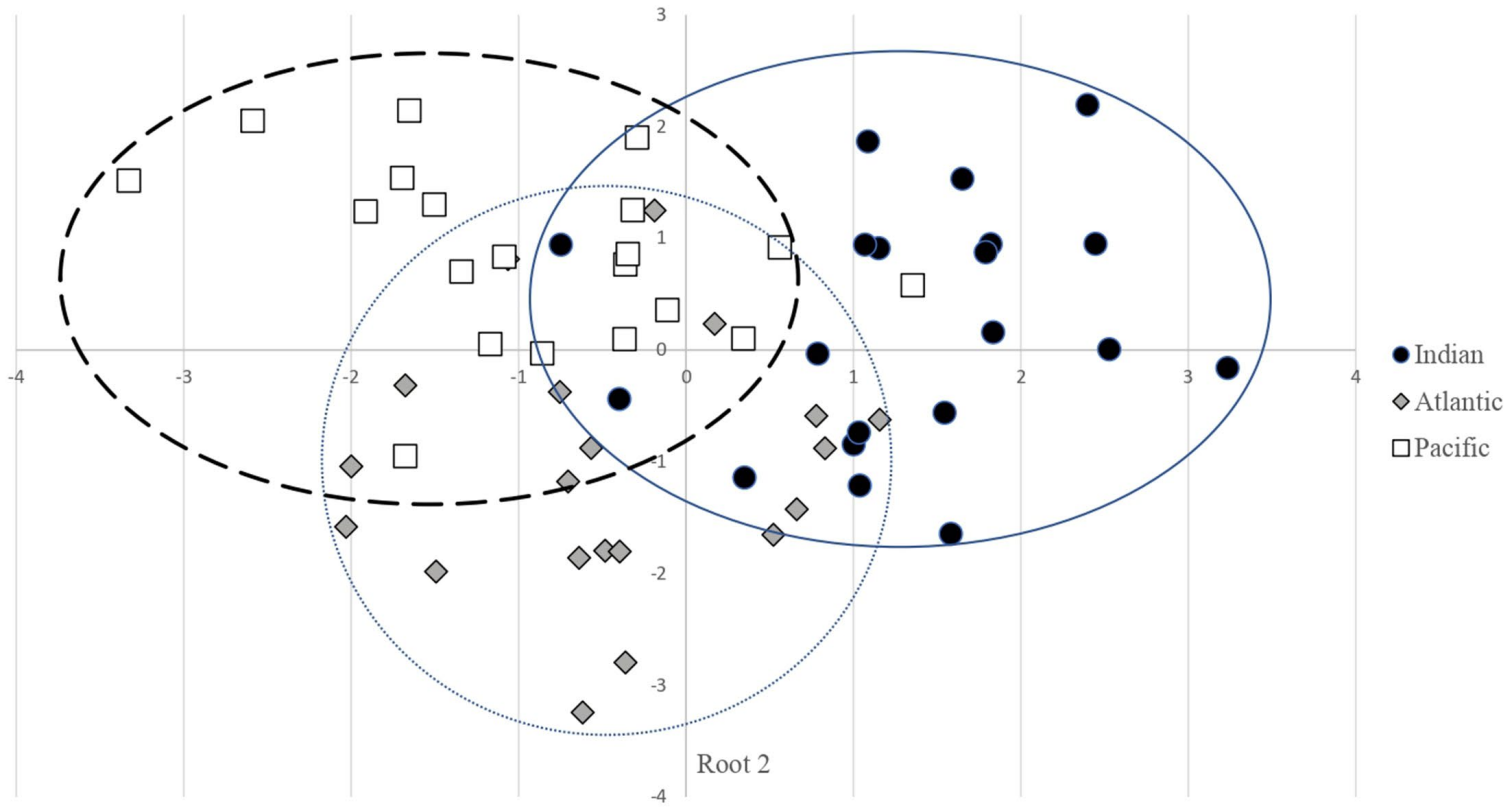
Plot of the discriminant functions (root 1 versus root 2) for classification of yellowfin tuna loins.

The two retained canonical functions maximized the variance among groups while minimizing the variance within groups. However, the corresponding biplot (Figure 2) was unable to achieve a distinct discrimination of samples according to oceanic origin. The Indian Ocean samples were predominantly found in the positive quadrants of both Roots, 50% of all samples were located and in the positive Root 1 and positive Root 2, while 40% of all samples were positioned in positive Root 1 and negative Root 2 quadrant. The tight clustering suggests relatively low within-group variability and high internal consistency in the amino acid profile of Indian tuna. The Pacific Ocean samples are mostly confined to the negative Root 1 and positive Root 2 quadrant (75% of all samples). This group also forms a compact cluster, reflecting a well-defined and homogeneous amino acid profile among Pacific samples. Notably, their separation from Indian samples along Root 1 and from Atlantic samples along both axes highlights the unique composition of this group. By contrast, the Atlantic Ocean samples exhibit greater dispersion, spanning both negative and near-zero values of Roots 1 and 2. This broader distribution reflects higher within-group variability, possibly indicating intermediate amino acid patterns or ecological diversity among the sampled individuals. Some degree of overlap with both Indian and Pacific samples is observed, suggesting shared biochemical features or transitional characteristics in their amino acid profiles.

The cross-validated classification matrix (Table 7) showed an overall classification accuracy of 71.7%, with accuracies of 65% for Atlantic and 75% for both Indian and Pacific loins. Of the 17 misclassified samples (28.3% of all), 41.2% were incorrectly assigned to the Indian Ocean, 35.3% to the Atlantic, and 23.5% to the Pacific. The overall misclassification rate observed in the Canonical Discriminant Analysis (28.3%) indicates that the discriminatory power of the model is moderate.

**Table 7:** Classification matrix of cross-validation for tuna origin (expressed as number of observations (%)) and accurate and total classification according the tuna origin

	Atlantic	Indian	Pacific	Total
Atlantic	13 (65.0%)	4 (20.0%)	3 (15.0%)	20 (100%)
Indian	4 (20.0%)	15 (75.0%)	1 (5.0%)	20 (100%)
Pacific	2 (10.0%)	3 (15.0%)	15 (75.0%)	20 (100%
Accurate classification	13 (21.7%)	15 (25.0%)	15 (25.0%)	43 (71.7%)
Total classification	19 (31.7%)	22 (36.7%)	19 (31.7%)	60 (100%)

Yellowfin tuna (YFT) origin did not significantly affect total protein content, which averaged 25.3 g/100 g of edible portion, allowing the product to bear a “high-protein” nutritional claim.

The compositional differences observed among samples from the Atlantic, Indian, and Pacific Oceans should be interpreted with caution. Factors such as diet, age, sex, and body size are known to influence amino acid profiles and the concentration of bioactive compounds in fish muscle. Because the specimens analysed in this study were obtained from commercial catches, detailed biological information for individual fish was not available. Consequently, the present study does not attempt to attribute the observed differences to specific biological, environmental, or genetic factors, but rather provides a comparative nutritional characterization of Yellowfin tuna from different oceanic regions.

## 4 Conclusion

YFT loins showed consistent total protein content (25.3 g/100 g of edible portion), supporting a ‘high-protein’ nutritional claim, and confirming that protein content and overall amino acid (AA) profile do not differ significantly among specimens from different oceans (H_01_). Likewise, muscle bioactive compounds were generally comparable across origins (H_02_), indicating that AA signatures and bioactive compound levels cannot reliably trace geographic origin (H_03_). However, ocean of origin significantly influenced individual AA profiles (*p* < 0.05), with differences observed in 11 of 17 quantified amino acids, including indispensable, conditionally indispensable, and dispensable AAs. These variations affected amino acid scores (AAS): Pacific YFT had no limiting AA, Atlantic YFT was limited in leucine, and Indian Ocean YFT showed the lowest profile, with valine (90.9%), isoleucine (89.0%), and leucine (70.4%) as limiting. Despite these differences, the essential amino acid index (EAAI) remained high across all samples, classifying them as ‘matching quality. YFT loins were also rich in bioactive compounds (anserine, carnosine, creatine) that support antioxidant and energy metabolism functions. While carnosine content was unaffected by origin, anserine and creatine were higher in Indian Ocean specimens. Canonical discriminant analysis (CDA) only partially separated samples by origin, achieving 71.7% overall classification accuracy, with 65% for both Atlantic and 75% for Indian and Pacific samples.

## Data Availability

The raw data supporting the conclusions of this article will be made available by the authors, without undue reservation.

## References

[1] FAO. Fishery and Aquaculture Statistics – Yearbook 2022 (2025). Available online at: https://openknowledge.fao.org/handle/20.500.14283/cd4312en (Accessed April 23, 2025)

[2] The Pew Charitable Trusts (2020). A global tuna valuation: netting billions 2020. Available online at: https://www.pewtrusts.org/en/research-and-analysis/reports/2020/10/netting-billions-2020-a-global-tuna-valuation (Accessed April 23, 2025)

[3] FAO. The State of World Fisheries and Aquaculture 2024. Rome: FAO (2024).

[4] Herpandi Huda N Rosma A Wan Nadiah WA. The tuna fishing industry: a new outlook on fish protein hydrolysates. Compr Rev Food Sci Food Saf (2011), 10:195–207. Available online at: https://onlinelibrary.wiley.com/doi/full/10.1111/j.1541-4337.2011.00155.x (Accessed October 1, 2025)

[5] EUMOFA. EU and Country Profiles Update 2025 [Internet] (2025). Available online at: https://eumofa.eu/eu-and-country-profiles-update-2025 (Accessed May 14, 2025)

[6] FAO. The State of World Fisheries and Aquaculture 2020. Sustainability in Action. Rome, Italy (2020). p. 224.

[7] Ariji M. Conjoint analysis of consumer preference for bluefin tuna. Fish Sci (2010) 76:1023–1028. Available online at: https://link.springer.com/article/10.1007/s12562-010-0297-4 (Accessed May 14, 2025)

[8] Jayedi A Shab-Bidar S Eimeri S Djafarian K. Fish consumption and risk of all-cause and cardiovascular mortality: a dose-response meta-analysis of prospective observational studies. Public Health Nutr (2018), 21:1297–1306. Available online at: https://pubmed.ncbi.nlm.nih.gov/29317009/ (Accessed August 30, 2025) 29317009 10.1017/S1368980017003834 PMC10261309

[9] Li F Liu X Zhang D. Fish consumption and risk of depression: a meta-analysis. J Epidemiol Community Health (2016) 70:299–304. Available online at: https://pubmed.ncbi.nlm.nih.gov/26359502/ (Accessed August 30, 2025) 26359502 10.1136/jech-2015-206278

[10] Godos J Micek A Currenti W Franchi C Poli A Battino M . Fish consumption, cognitive impairment and dementia: an updated dose-response meta-analysis of observational studies. Aging Clin Exp Res (2024) 36. Available online at: https://pubmed.ncbi.nlm.nih.gov/39162889/ (Accessed August 30, 2025) 10.1007/s40520-024-02823-6 PMC11335789 39162889

[11] Zhao LG Sun JW Yang Y Ma X Wang YY Xiang YB. Fish consumption and all-cause mortality: a meta-analysis of cohort studies. Eur J Clin Nutr (2016), 70:155–161. Available online at: https://www.nature.com/articles/ejcn201572 (Accessed August 30, 2025) 25969396 10.1038/ejcn.2015.72

[12] EUMOFA. The EU Fish Market 2021 Edition is Now Online (2022). Available online at: https://ec.europa.eu/oceans-and-fisheries/news/eu-fish-market-2021-edition-now-online-2021-11-22_pt (Accessed November 27, 2021)

[13] NFI. Top 10 List for Seafood Consumption - About Seafood (2020). Available online at: https://aboutseafood.com/about/top-ten-list-for-seafood-consumption/ (Accessed September 26, 2021)

[14] Jumilla-Lorenz D Briones T Parés D. Physicochemical and sensory characterization of raw tuna muscle for plant-based fish analogs development purposes. Heliyon (2024), 10. Available online at: https://pubmed.ncbi.nlm.nih.gov/39430479/ (Accessed May 14, 2025) 10.1016/j.heliyon.2024.e38749 PMC11489372 39430479

[15] Peng S Chen C Shi Z Lu W. Amino acid and fatty acid composition of the muscle tissue of yellowfin tuna (*Thunnus albacares*) and bigeye tuna (*Thunnus obesus*). J Food Nutr Res. (2013) 1:42–5. doi: 10.12691/jfnr-1-4-2

[16] Salinas D Sánchez H Gallegos L Moreno M Pérez L Salazar D Undervalued tuna meat (*Thunus obesus* and *Katsuwonus pelamis lineaus*) to develop sausages. Vitae (2024) 31:1–10. Available online at: http://www.scielo.org.co/scielo.php?script=sci_arttext&pid=S0121-40042024000100003&lng=en&nrm=iso&tlng=en (Accessed May 16, 2025)

[17] Triyastuti MS Wijaya N Kurnia Dewi L Indah Budiarti G. Nutrient content and sensory characteristics of tuna fish Dimsum (yellowfin). E3S Web Conf (2023), 448:01002. Available online at: https://www.e3s-conferences.org/articles/e3sconf/abs/2023/85/e3sconf_icenis2023_01002/e3sconf_icenis2023_01002.html (Accessed May 14, 2025)

[18] Nakamura YN Ando M Seoka M Kawasaki K i Tsukamasa Y. Changes of proximate and fatty acid compositions of the dorsal and ventral ordinary muscles of the full-cycle cultured Pacific bluefin tuna *Thunnus orientalis* with the growth. Food Chem. (2007) 103:234–41. doi: 10.1016/j.foodchem.2006.07.064

[19] Sasidharan A Rustad T Cusimano GM. Tuna sidestream valorization: a circular blue bioeconomy approach. Environ Sci Pollut Res (2023) 31:62230–62248. Available online at: https://link.springer.com/article/10.1007/s11356-023-28610-w 10.1007/s11356-023-28610-w PMC11606988 37434051

[20] Tan YL Juhari NH Jambari NN Rozzamri A Nor-Khaizura MAR Ismail-Fitry MR (2025). Tuna and tuna products: a review of the nutrition, processing, safety, and future prospects. Fish Sci 91:1–22. Available online at: https://link.springer.com/article/10.1007/s12562-025-01878-2 (Accessed May 14, 2025)

[21] Domingues VF Quaresma M Sousa S Rosas M Ventoso B Nunes ML . Evaluating the lipid quality of yellowfin tuna (*Thunnus albacares*) harvested from different oceans by their fatty acid signatures. Foods. (2021) 10:2816–2824. doi: 10.3390/foods10112816, 34829097 PMC8620007

[22] Banaszak M Dobrzyńska M Kawka A Górna I Woźniak D Przysławski J . Role of Omega-3 fatty acids eicosapentaenoic (EPA) and docosahexaenoic (DHA) as modulatory and anti-inflammatory agents in noncommunicable diet-related diseases – reports from the last 10 years. Clin Nutr ESPEN. (2024) 63:240–58. doi: 10.1016/j.clnesp.2024.06.053, 38980796

[23] FAO. World Review of Highly Migratory Species and Straddling Stocks, vol. 70. Rome, Italy (1994).

[24] Quaresma MAG Pereira G Nunes ML Sponda C Jardim A Gonçalves H . Evaluating dried salted cod amino acid signature for nutritional quality assessment and discriminant analysis. Front Nutr. (2023) 10 10.3389/fnut.2023.1144713 PMC10140297 37125032

[25] Wu G. "Functional Amino Acids in Nutrition and Health". In: Amino Acids. New York, USA (2013) p. 407–11. doi: 10.1007/s00726-013-1500-6 23595206

[26] Wu G. Amino Acids: Metabolism, Functions, and Nutrition. In: Amino Acids. New York, USA (2009) p. 1–17. 10.1007/s00726-009-0269-0 19301095

[27] Wu G (2020). Important roles of dietary taurine, creatine, carnosine, anserine and 4-hydroxyproline in human nutrition and health. Amino Acids, 52:329–360. Available online at: https://link.springer.com/article/10.1007/s00726-020-02823-6 (Accessed March 6, 2026) 32072297 10.1007/s00726-020-02823-6 PMC7088015

[28] Kreider RB Stout JR. Creatine in health and disease. Forum Nutr, (2021) 13:1–28. Available online at: https://www.mdpi.com/2072-6643/13/2/447 (Accessed March 7, 2026) 10.3390/nu13020447 PMC7910963 33572884

[29] AOAC. Official methods of analysis, 21st edition (2019) - AOAC INTERNATIONAL (2019). Available online at: https://www.aoac.org/official-methods-of-analysis-21st-edition-2019/ (Accessed December 1, 2021)

[30] Aristoy MC Toldrá F. Deproteinization techniques for HPLC amino acid analysis in fresh pork muscle and dry-cured ham. J Agric Food Chem. (1991) 39:1792–5. doi: 10.1021/jf00010a020

[31] Quaresma MAG Antunes IC Ferreira BG Parada A Elias A Barros M . The composition of the lipid, protein and mineral fractions of quail breast meat obtained from wild and farmed specimens of common quail (*Coturnix coturnix*) and farmed Japanese quail (*Coturnix japonica* domestica). Poult Sci. (2022) 101:101505. doi: 10.1016/j.psj.2021.101505, 34818612 PMC8626699

[32] Roseiro LC Santos C Gonçalves H Moniz C Afonso I Tavares M . Concentration of antioxidants in two muscles of mature dairy cows from Azores. Meat Sci. (2014) 96:870–5. doi: 10.1016/j.meatsci.2013.09.005, 24211545

[33] WHO. Protein and amino acid requirements in human nutrition: report of a joint FAO/WHO/UNU expert consultation. World Health Organisation, technical report series, no 935 (2007). Available online at: https://www.sochob.cl/pdf/libros/Dietary%20protein%20quality%20evaluation%20in%20human%20nutrition.pdf (Accessed March 13, 2022)

[34] Murphy R McConell G Cameron-Smith D Watt K Ackland L Walzel B Creatine transporter protein content, localization, and gene expression in rat skeletal muscle. Am J Physiol Cell Physiol (2001) 280. Available online at: https://journals.physiology.org/doi/10.1152/ajpcell.2001.280.3.C415 (Accessed July 25, 2025) 10.1152/ajpcell.2001.280.3.C415 11171559

[35] Özyurt G Polat A. Amino acid and fatty acid composition of wild sea bass (*Dicentrarchus labrax*): a seasonal differentiation. Eur Food Res Technol (2006), 222:316–320. Available online at: https://link.springer.com/article/10.1007/s00217-005-0040-z (Accessed May 14, 2025)

[36] European Union. Regulation - 1924/2006 - EN - EUR-lex (2006). Available online at: https://eur-lex.europa.eu/legal-content/EN/TXT/?uri=CELEX:32006R1924 (Accessed September 17, 2025)

[37] Biji K. B. Kumari K. R. R. Anju K. A. Mathew S. Ravishankar C. N. Quality characteristics of yellowfin tuna (*Thunnus albacares*) in the fish landing Centre at Cochin, India. Fish Technol (2016), 53:313–319. Available online at: http://hdl.handle.net/123456789/2443 (Accessed May 26, 2025)

[38] WHO. Protein and Amino Acid Requirements in Human Nutrition: Report of a Joint WHO/FAO/UNU Expert Consultation. Geneva: World Health Organization (WHO) (2002). 18330140

[39] Standard Tables of Food Composition In Japan – 2015 (2015). Available online at: https://www.mext.go.jp/en/policy/science_technology/policy/title01/detail01/sdetail01/1388553.htm (Accessed April 8, 2026)

[40] Lozano G Hardisson A. "Fish as food". In: Caballero B Trugo L Finglas P, editors. Encyclopedia of Food Sciences and Nutrition, 2nd Edn San Diego, California (USA): Academic Press (2003). p. 2417–23.

[41] Bahurmiz O. M. Proximate and fatty acid composition of three tuna species from Hadhramout coast of the Arabian Sea, Yemen. Hadhramout Univ J Nat Appl Sci (2019), 16:63–71. Available online at: https://hu.edu.ye/hu-publications/wp-content/uploads/2021/01/Proximate-and-fatty-acid-composition-of-three-tuna-species-from-Hadhramout-coast-of-the-Arabian-Sea-YemenP_16_1_2019.pdf (Accessed 19 March, 2026).

[42] Wang W. Wu Z. Dai Z. Yang Y. Wang J. Wu G. Glycine metabolism in animals and humans: implications for nutrition and health. Amino Acids (2013) 45:463–477. Available online at: https://link.springer.com/article/10.1007/s00726-013-1493-1 (Accessed 19 March, 2026). 23615880 10.1007/s00726-013-1493-1

[43] FAO. Quality and quality changes in fresh fish - 4. Chemical composition. In: FAO fisheries technical paper - 348 (1995). Available online at: https://www.fao.org/4/V7180E/v7180e05.htm?utm_source=chatgpt.com (Accessed March 5, 2026)

[44] McLean E Alfrey KB Gatlin DM Gibson Gaylord T Barrows FT. Muscle amino acid profiles of eleven species of aquacultured animals and their potential value in feed formulation. Aquac Fish. (2024) 9:642–52. doi: 10.1016/j.aaf.2022.04.010

[45] Zlatanos S. Laskaridis K. Feist C. Sagredos A. Proximate composition, fatty acid analysis and protein digestibility-corrected amino acid score of three Mediterranean cephalopods. Mol Nutr Food Res. (2006), 50:967–970. Available online at: https://onlinelibrary.wiley.com/doi/full/10.1002/mnfr.200600003 (Accessed May 15, 2025) 17009215 10.1002/mnfr.200600003

[46] Ünal-Şengör GF Yildiz M Metin Ö Ofori-Mensah S Ceylan Z. Compositions of gilthead sea bream (*Sparus aurata* Linnaeus, 1758) from different culture systems. Aquac Int (2025), 33:1–19. Available online at: https://link.springer.com/article/10.1007/s10499-024-01735-6 (Accessed May 16, 2025)

[47] Talib A Tangke U Saharin MD Nurhidayati F. Extraction of collagen bone fish (*Thunnus albacares*) into gelatin with CH3COOH treatment. Open Access Res J Biol Pharm (2022) 4:015–021. Available online at: https://oarjbp.com/content/extraction-collagen-bone-fish-thunnus-albacares-gelatin-ch3cooh-treatment (Accessed July 5, 2025)

[48] Ochiai Y Ozawa H. Biochemical and physicochemical characteristics of the major muscle proteins from fish and shellfish. Fish Sci (2020), 86:729–740. Available online at: https://link.springer.com/article/10.1007/s12562-020-01444-y (Accessed July 12, 2025)

[49] Sabu S. "Introduction to fish proteins". In: Fish Structural Proteins and Its Derivatives: Functionality and Applications Duxford, Cambridge, UK (2024). p. 3–17.

[50] Musyl MK Brill RW Boggs CH Curran DS Kazama TK Seki MP. Vertical movements of bigeye tuna (*Thunnus obesus*) associated with islands, buoys, and seamounts near the main Hawaiian islands from archival tagging data. Fish Oceanogr (2003) 12:152–169. Available online at: https://onlinelibrary.wiley.com/doi/full/10.1046/j.1365-2419.2003.00229.x (Accessed September 19, 2025)

[51] Barth JMI Damerau M Matschiner M Jentoft S Hanel R. Genomic differentiation and demographic histories of Atlantic and Indo-Pacific yellowfin tuna (*Thunnus albacares*) populations. Genome Biol Evol (2017) 9:1084–1098. Available online at: https://pubmed.ncbi.nlm.nih.gov/28419285/ (Accessed June 23, 2025) 28419285 10.1093/gbe/evx067 PMC5408087

[52] Díaz-Jaimes P Uribe-Alcocer M. Spatial differentiation in the eastern Pacific yellowfin tuna revealed by microsatellite variation. Fish Sci (2006), 72:590–596. Available online at: https://link.springer.com/article/10.1111/j.1444-2906.2006.01188.x (Accessed June 23, 2025)

[53] Kunal SP Kumar G Menezes MR Meena RM. Mitochondrial DNA analysis reveals three stocks of yellowfin tuna *Thunnus albacares* (Bonnaterre, 1788) in Indian waters. Conserv Genet (2013) 14:205–213. Available online at: https://link.springer.com/article/10.1007/s10592-013-0445-3 (Accessed June 23, 2025)

[54] Xipsiti M. Protein quality evaluation: FAO perspective. Front Nutr. (2024) 11:1446879. doi: 10.3389/fnut.2024.1446879, 39698253 PMC11654912

[55] Jukić I Kolobarić N Stupin A Matić A Kozina N Mihaljević Z . Carnosine, small but mighty—prospect of use as functional ingredient for functional food formulation. Antioxidants (2021), 10:1037. Available online at: https://www.mdpi.com/2076-3921/10/7/1037/htm (Accessed June 25, 2025) 34203479 10.3390/antiox10071037 PMC8300828

[56] Kulczyński B Sidor A Gramza-Michałowska A. Characteristics of selected antioxidative and bioactive compounds in meat and animal origin products. Antioxidants (2019), 8:335. Available online at: https://www.mdpi.com/2076-3921/8/9/335/htm 31443517 10.3390/antiox8090335 PMC6769838

[57] Hill CA Harris RC Kim HJ Harris BD Sale C Boobis LH . (2007). Influence of beta-alanine supplementation on skeletal muscle carnosine concentrations and high intensity cycling capacity. Amino Acids, 32:225–233. Available online at: https://pubmed.ncbi.nlm.nih.gov/16868650/16868650 10.1007/s00726-006-0364-4

[58] Mannion AF Jakeman PM Dunnett M Harris RC PLT Willan (1992). Carnosine and anserine concentrations in the quadriceps femoris muscle of healthy humans. Eur J Appl Physiol Occup Physiol 64:47–50. Available online at: https://link.springer.com/article/10.1007/BF00376439 (Accessed July 25, 2025) 1735411 10.1007/BF00376439

[59] Kalbe C Metzger K Gariépy C Palin MF. Effect of muscle fibre types and carnosine levels on the expression of carnosine-related genes in pig skeletal muscle. Histochem Cell Biol. (2023) 160:63–77. doi: 10.1007/s00418-023-02193-6, 37171629 PMC10313551

[60] Barbaresi S Maertens L Claeys E Derave W De Smet S. Differences in muscle histidine-containing dipeptides in broilers. J Sci Food Agric (2019) 99:5680–5686. Available online at: https://pubmed.ncbi.nlm.nih.gov/31150113/ (Accessed July 25, 2025) 31150113 10.1002/jsfa.9829

[61] Lu Z Li Q Yongo E Xiao J Guo Z. Comparative energy metabolism in red and white muscles of juvenile yellowfin tuna, *Thunnus albacore*. Front Mar Sci (2025) 12:1585044. Available online at: http://daehwankimlab.github.io/hisat2/ (Accessed July 11, 2025)

[62] Abe H Brill RW Hochachka PW (1986). Metabolism of L-histidine, carnosine, and anserine in skipjack tuna. 59:439–450. Available online at: https://www.journals.uchicago.edu/doi/10.1086/physzool.59.4.30158597 (Accessed July 27, 2025)

[63] Bodin N Amiel A Fouché E Sardenne F Chassot E Debrauwer L . NMR-based metabolic profiling and discrimination of wild tropical tunas by species, size category, geographic origin, and on-board storage condition. Food Chem. (2022) 371:131094 34583182 10.1016/j.foodchem.2021.131094

